# Rapid or Immediate ART, HIV Stigma, Medical Mistrust, and Retention in Care: An Exploratory Mixed Methods Pilot Study

**DOI:** 10.1007/s10461-023-04058-4

**Published:** 2023-04-18

**Authors:** Ofole Mgbako, Claire Loughran, Rachel Mathu, Delivette Castor, Jacob McLean, Magdalena E. Sobieszczyk, Susan Olender, Peter Gordon, Javier Lopez-Rios, Robert H. Remien

**Affiliations:** 1 Division of Infectious Diseases and Immunology, Department of Internal Medicine, NYU Langone Health, New York, NY USA; 2 NYU Langone Institute for Excellence in Health Equity, New York, NY USA; 3 NewYork Presbyterian, Columbia University Irving Medical Center, New York, NY USA; 4 ICAP, Columbia University Mailman School of Public Health, New York, NY USA; 5 Division of Infectious Disease, Department of Internal Medicine, Columbia University Irving Medical Center, New York, NY USA; 6 Department of Community Health and Prevention, Dornsife School of Public Health, Drexel University, Philadelphia, PA USA; 7 HIV Center for Clinical and Behavioral Studies, NY State Psychiatric Institute and Columbia University, New York, NY USA; 8 NYC Health + Hospitals/Bellevue, 462 1st Ave. H building, Office 16s10, New York, NY 10016 USA

**Keywords:** Rapid ART, Immediate ART, Medical mistrust, Stigma, Retention in care, HIV

## Abstract

Rapid or immediate antiretroviral therapy (iART) after HIV diagnosis improves linkage to care and time to viral suppression. However, iART may affect or be affected by HIV-related stigma and medical mistrust. In this mixed-methods pilot study, we examined the bi-directional role of HIV stigma, medical mistrust, and visit adherence (VA) in the context of iART in a diverse, newly diagnosed patient population. Participants were recruited from an HIV clinic in New York City and we utilized a convergent parallel design integrating quantitative data from demographic surveys, the HIV Stigma Survey (HIVSS), the Medical Mistrust Index (MMI) and electronic medical records, and qualitative data from in-depth interviews. Among the sample (N = 30), 26% (N = 8) initiated ART same-day or within 3 days, while the majority (N = 17) initiated between 4 and 30 days, and 17% (N = 5) initiated ART > 30 days. The median (range) age was 35, and most were English-speaking, Black or Hispanic men and identified as gay. Time to ART initiation was associated with time to linkage to care and time to viral suppression. Day 0–3 group’s major theme was iART as stigma prevention, and they had the highest mean HIVSS, lowest MMI score, and a visit adherence of 0.86. Day 4–30 group’s major theme was alleviation of internalized stigma, and they had the lowest mean HIVSS score, and highest visit adherence of 0.91. Day > 30 group’s major theme was exacerbation of perceived or anticipated stigma, had the highest MMI score and a visit adherence of 0.85. iART implementation requires equitable strategies that address HIV-stigma and mistrust.

**Supplementary Information:**

The online version contains supplementary material available at 10.1007/s10461-023-04058-4.

## Introduction

Rapid or immediate antiretroviral therapy (iART) upon HIV diagnosis has become standard of care globally, including the United States (US) [1–3]. iART is defined as the offer of ART on the same day or as soon as possible after HIV diagnosis without waiting for laboratory testing, and includes various time frames but generally considered within 30 days. Global randomized controlled trials and observational studies in various clinical settings in the U.S have established that iART improves linkage to care and shortens time to viral suppression [4–7]. The HIV epidemic in the US continues to be marked by persistent inequities by race, ethnicity, gender, and sexual minority status; it is critical to understand the psychosocial costs and benefits of iART among these groups during a highly vulnerable time immediately post-diagnosis [8, 9].

HIV-related stigma has influenced care engagement on multiple levels for the most affected populations living with HIV in the US [10–12]. Whether internalized, anticipated, or enacted stigma from prior lived experiences, studies have shown that racial, gender and sexual minorities experience higher rates of various forms of HIV-related stigma, leading to suboptimal HIV care outcomes [13, 14]. Medical mistrust is another barrier to HIV care engagement and ART adherence [15]. Studies have shown that people with HIV (PWH) believe there is an overemphasis on ART at the expense of other non-HIV related priorities—a consequence of distrust in providers and the health system [16]. Therefore, it is vital to understand *perceptions and experiences* of receiving iART, and how receiving iART may impact HIV-related stigma and medical trust/mistrust, and how that, in turn, influences HIV care engagement.

Few qualitative studies have explored the impact of iART during the post-diagnosis period for those newly diagnosed with HIV in the US. A recent qualitative study from San Diego among 19 men who have sex with men (MSM) with HIV showed that patients’ desires to remain virally suppressed and prevent ongoing viral transmission were powerful motivators in the acceptance of iART [17]. However, no studies examine more deeply how iART impacts important psychosocial factors such as HIV-related stigma and medical trust/mistrust at the time of diagnosis and the subsequent effect on HIV care engagement. This mixed-methods pilot study sought to understand the psychosocial costs and benefits of iART for a newly diagnosed predominantly Black, Latino, and MSM patient population in New York City and explore associations between iART, HIV-related stigma, medical trust/mistrust, and retention in care.

## Methods

### Study Design

**Fig. 1:**
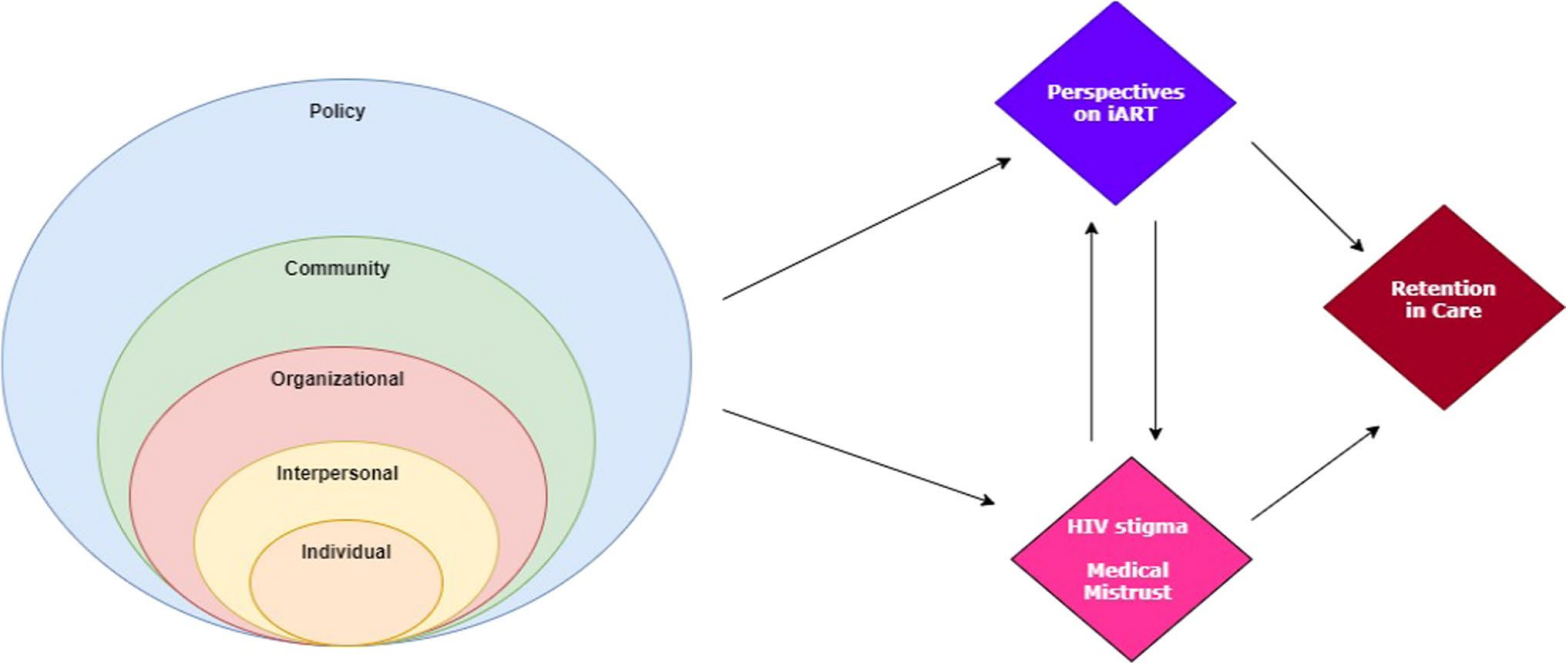
Conceptual framework integrating social-ecological model and theory of planned behavior

We utilized a convergent parallel mixed-methods research design, combining quantitative data from a demographic survey, stigma and mistrust surveys, and electronic medical record (EMR); and qualitative data from semi-structured in-depth interviews (IDIs). Theoretical considerations regarding the role of HIV-related stigma and medical mistrust were grounded in a conceptual framework encompassing the socio-ecological model and the theory of planned behavior, underlining the multi-level influences that determine the impact of iART, HIV stigma and medical trust/mistrust on retention in care (Fig. 1).

### Participants and Eligibility Criteria

Participants were recruited from an HIV care clinic in an academic medical center in New York City, serving a safety net population between October 2020 and June 2021. The inclusion criteria were: [1] aged 18 years or older; [2] diagnosed with HIV on or after January 1, 2018; [3] offered iART; [4] enrolled in the clinic at the medical center for at least 6 months; [5] reside within one of the NYC boroughs; and [6] English or Spanish speaking.

### Procedures

Participants were recruited in-person, by phone, or by responding to flyers posted in clinic waiting areas. Participants were electronically consented via REDCap. EMR data were abstracted. The interview guide and surveys were developed to last 60–90 min, translated by a native Spanish speaker, and back-translated into English. The interview and surveys were administered either in-person or virtually while maintaining participant privacy and confidentiality. All interviews were audio-recorded, professionally transcribed verbatim and professionally translated from Spanish to English. Participants received $50 gift cards.

### Qualitative Measures

#### Qualitative Interview Guide

IDIs were conducted using a semi-structured interview guide, iteratively developed by the research team and grounded in the conceptual framework. The interview guide was piloted with the first two study participants (included in the final analysis), and adjustments were made to ensure the meaning and intention of questions were understood. The five domains of the guide were [1] Experiences and perspectives from new HIV diagnosis to iART initiation; [2] HIV care engagement; [3] Medical trust/mistrust; [4] Stigma; and [5] COVID-19.

### Quantitative Measures

#### iART

The cohort was divided into three groups according to time to ART initiation: 0–3 days, 4–30 days, and > 30 days. This categorization is based on the New York State Department of Health AIDS Institute definition of rapid ART initiation, which states “ideally on the same day or within 72 hours” however never beyond 30 days of diagnosis [18].

#### Participant Demographic Survey

Using a structured instrument developed by the research team, participant demographics collected included age, country of birth, primary language spoken, race/ethnicity, gender identity, sexual orientation, the highest level of education, current employment status, current housing situation, current annual income, health insurance and relationship status.

#### HIV Stigma Survey (HIVSS)

A validated 40-item instrument was used to examine participants’ internalized, interpersonal and community-based HIV stigma [19]. Each item was scored on a 4-point Likert-type scale: 1 = Strongly Disagree, 2 = Disagree, 3 = Agree, 4 = Strongly Agree. The HIVSS is comprised of 4 subscales addressing different dimensions of stigma: negative self-image, personalized stigma, public attitudes about HIV and diagnosis disclosure. We interpreted the type of HIV-related stigma (e.g. internalized, anticipated, enacted, community) according to the framework laid out in Turan et al. [12]. Negative self-image corresponds to internalized stigma, while personalized stigma corresponds to anticipated, perceived, or enacted stigma. Public attitudes correspond to community stigma.

#### Medical Mistrust Index (MMI)

A validated 17-item instrument designed to measure individual trust/mistrust in the health care system was used. The Medical Mistrust Index (MMI) is not particular to one provider at a healthcare facility (also known as “provider trust”), but rather healthcare institutions [20]. Each item was scored on a 4-point Likert-type scale: 1 = Strongly Disagree, 2 = Disagree, 3 = Agree, 4 = Strongly Agree.

#### Medical Chart Review

We conducted an EMR review of demographic and clinical outcomes over the first 6 months after diagnosis. Participant demographics included age, race/ethnicity, gender identity, sexual orientation, language, and health insurance. HIV risk factors (heterosexual, MSM, IDU, blood transfusion, etc.) and clinical outcomes were recorded, including number of comorbidities, dates and locations of HIV diagnosis and iART offer/initiation, and date of initial viral suppression, if ever. Calculations were derived from the date of events to establish time (in days) to linkage to care, offer of iART, initiation of iART, and viral suppression. Retention in care, or visit adherence, was gathered 6 months post-iART (number of completed HIV medical care visits over number of scheduled visits) [21].

### Data Analysis

Data from the demographic survey and EMR were analyzed using descriptive statistics. HIVSS and MMI were analyzed using bar graphs and box plots. Fisher’s exact test was used to examine differences between categories of time to ART initiation. Mann Whitney U test was used to analyze difference between median and ANOVA was used to analyze difference between means. In order to limit misleading results due to a small cohort, continuous variables were assessed for skewness to determine whether the median (range) or mean (standard deviation) was more appropriate to report. If the results were skewed, median results (range) were reported. For variables following a normal distribution, mean results (standard deviation) were reported. For the HIVSS and MMI scores, we used mean (standard deviation) because this has been the traditional measure reported in the literature. We also included a sensitivity analysis breaking down the iART groups as following: 0–7 days, 8–30 days, > 30 days.

#### Qualitative Analysis

Using an inductive approach, OM and CL developed an initial codebook using *a priori* codes derived from the interview guide and the conceptual framework. Emergent codes were also identified. Each researcher independently coded a set of transcripts and inconsistencies were reconciled among the larger research team. We then calculated inter-coder agreement (pooled Kappa of 0.91). We used Dedoose to analyze data, looking for code co-occurrence pertinent to our research question. Each quote is contextualized by the iART group, and the race and ethnicity answered by participants in the demographic survey. Other demographics were not included with each quote to protect the identity of the participants.

### Data Triangulation and Integration

Qualitative data were triangulated with survey and EMR data to enhance quality. For example, if a participant’s race was marked as “Other” in chart review, their demographic survey and in-depth interview responses were used to clarify their race. Quantitative (HIVSS, MMI scores, and visit adherence) and qualitative data (themes from in-depth discussion) were then combined to determine convergence and divergence of the data in an integrated table.

## Results

### Quantitative Results

Of the 30 enrolled participants, 20 conducted the study virtually, and 10 conducted the study in-person. Four interviews were conducted in Spanish and 26 in English. Table 1 shows the overall demographic characteristics and of each group by time to ART initiation. The overall cohort (N = 30) had a mean (SD) age of 35.4 (11.7), was mostly Black (33%) or Hispanic (60%), English-speaking (73%), gay (53%), male (80%), had at least some college (73%), making less than $20,000 (43%), stably housed (93%), and on Medicaid (60%). The 4–30 day group reported a higher income.

**Table 1:** Demographic characteristics of participants (N = 30)

Characteristic	Time to ART initiation (N = 30)				
	0–3 (N = 8)	4–30 (N = 17)	> 30 (N = 5)	Total (N = 30)	p value
	N (%)	N (%)	N (%)	N (%)	
Age					0.713
Mean (SD)	34.8 (8.7)	34.5 (9.3)	39.4 (22.1)	35.4 (11.6)	
Country of birth					1.000
United States/territory	6 (75.0)	13 (76.5)	4 (80.0)	23 (76.7)	
Other	2 (25.0)	4 (23.5)	1 (20.0)	7 (23.3)	
Primary language spoken					0.730
English	7 (87.5)	12 (70.6)	3 (60.0)	22 (73.3)	
Spanish	1 (12.5)	4 (23.5)	2 (40.0)	7 (23.3)	
Other	0 (0.0)	1 (5.9)	0 (0.0)	1 (3.3)	
Race^a^					0.776
Black/African American	4 (50.0)	4 (23.5)	2 (40.0)	10 (33.3)	
Native American/Alaskan Native	0 (0.0)	1 (5.9)	0 (0.0)	1 (3.3)	
White	0 (0.0)	3 (17.7)	0 (0.0)	3 (10.0)	
Other	4 (50.0)	9 (52.9)	3 (60.0)	16 (53.3)	
Ethnicity					1.000
Hispanic	5 (62.5)	10 (58.8)	3 (60.0)	18 (60.0)	
Non-hispanic	3 (37.5)	7 (41.2)	2 (40.0)	12 (40.0)	
Gender identity					0.813
Man	6 (75.0)	13 (76.5)	5 (100)	24 (80.0)	
Woman	2 (25.0)	3 (17.7)	0 (0.0)	5 (16.7)	
Transgender woman	0 (0.0)	1 (5.9)	0 (0.0)	1 (3.3)	
Sexual orientation					0.892
Heterosexual/straight	3 (37.5)	4 (23.5)	3 (60.0)	10 (33.3)	
Bisexual	0 (0.0)	2 (11.8)	0 (0.0)	2 (6.7)	
Gay	5 (62.5)	9 (52.9)	2 (40.0)	16 (53.3)	
Queer	0 (0.0)	1 (5.9)	0 (0.0)	1 (3.3)	
Unsure/questioning	0 (0.0)	1 (5.9)	0 (0.0)	1 (3.3)	
Highest level of education					0.656
Some high school	1 (12.5)	1 (5.9)	0 (0.0)	2 (6.7)	
High School Diploma/GED	0 (0.0)	4 (22.5)	2 (40.0)	6 (20.0)	
Associate’s Degree/some college	4 (50.0)	6 (35.3)	2 (40.0)	12 (40.0)	
Bachelor’s Degree or Higher	3 (37.5)	6 (35.3)	1 (20.0)	10 (33.3)	
Employment status					0.156
Employed/self-employed	4 (50.0)	9 (52.9)	1 (20.0)	14 (46.7)	
Unemployed/unable to work	3 (37.5)	8 (47.1)	2 (40.0)	13 (43.3)	
Student/retired	1 (12.5)	0 (0.0)	2 (40.0)	3 (10.0)	
Housing situation					1.000
Stably housed	7 (87.5)	16 (94.1)	5 (100)	28 (93.3)	
Unstably housed	1 (12.5)	1 (5.9)	0 (0.0)	2 (6.7)	
Current annual income					0.052
<$20,000	5 (62.5)	5 (29.4)	3 (60.0)	13 (43.3)	
$20,000–39,999	1 (12.5)	3 (17.7)	2 (40.0)	6 (20.0)	
$40,000–59,999	0 (0.0)	8 (47.1)	0 (0.0)	8 (26.7)	
>$60,000	2 (25.0)	1 (5.9)	0 (0.0)	3 (10.0)	
Health insurance^b^					0.379
ADAP	2 (25.0)	1 (5.9)	0 (0.0)	3 (10.0)	
Medicaid/medicare	5 (62.5)	9 (52.9)	4 (80.0)	18 (60.0)	
Private	1 (12.5)	7 (41.2)	1 (20.0)	9 (30.0)	
Marital status					0.515
Never married	6 (75.0)	11 (64.7)	2 (40.0)	19 (63.3)	
Divorced/separated/other	2 (25.0)	6 (35.3)	3 (60.0)	11 (36.7)	

^a^Preference was given to patient survey responses over chart review when determining race and ethnicity. All responses of “Other” in which “Latino,” Hispanic,” or some variant were entered were maintained as “Other” as per NIH guidelines (patients also responded “Puerto Rican” “Spanish” and “Dominican”; one patient said “mixed” and one said “biracial”)

^b^PWH who are uninsured or underinsured in New York state are eligible for immediate coverage via ADAP (AIDS Drug Assistance Program). Patients who present to our site without insurance are enrolled immediately, so no patient in this cohort is listed as uninsured

Table 2 shows clinical characteristics, HIVSS and MMI scores, and HIV outcomes. The overall cohort identified MSM sexual contact as their major HIV risk factor (63%), had less than 2 comorbidities (80%), and about a quarter identified either a substance use (23%) or mental health diagnosis (27%). About 20% had a CD4 less than 200 at presentation. The > 30-day group had more individual participants reporting 3 or more comorbidities.

**Table 2:** Clinical characteristics of participants (N = 30)

Characteristic	Time to ART initiation (Days) (N = 30)				
	0–3 (N = 8)	4–30 (N = 17)	> 30 (N = 5)	Total (N = 30)	p value
	N (%)	N (%)	N (%)	N (%)	
HIV risk factor					0.175
Heterosexual sex	3 (37.5)	4 (23.5)	2 (40.0)	9 (30.0)	
MSM	4 (50.0)	13 (76.5)	2 (40.0)	19 (63.3)	
MSM + IDU	1 (12.5)	0 (0.0)	0 (0.0)	1 (3.3)	
IDU	0 (0.0)	0 (0.0)	1 (20.0)	1 (3.3)	
Number of comorbidities^a^					0.020
0–2	7 (87.5)	15 (88.2)	2 (40.0)	24 (80.0)	
3–5	0 (0.0)	2 (11.8)	3 (60.0)	5 (16.7)	
6+	1 (12.5)	0 (0.0)	0 (0.0)	1 (3.3)	
Mental health diagnosis					0.137
Yes	1 (12.5)	7 (41.2)	0 (0.0)	8 (26.7)	
No	7 (87.5)	10 (58.8)	5 (100)	22 (73.3)	
Substance use diagnosis					0.349
Yes	3 (37.5)	4 (23.5)	0 (0.0)	7 (23.3)	
No	5 (62.5)	13 (76.5)	5 (100)	23 (76.7)	
Initial CD4					1.000
> 200	7 (87.5)	13 (76.5)	4 (80.0)	24 (80.0)	
< 200	1 (12.5)	4 (23.5)	1 (20.0)	6 (20.0)	
Initial viral load					0.507
< 100,000	5 (62.5)	6 (35.3)	3 (60.0)	14 (46.7)	
100–500,000	1 (12.5)	6 (35.3)	2 (40.0)	9 (30.0)	
> 500,000	2 (25.0)	5 (29.4)	0 (0.0)	7 (23.3)	
Days to linkage to care median (range)	4.0 (0.0–67.0)	6.0 (1.0–210.0)	146.0 (35.0–163.0)	9.0 (0.0–210.0)	0.006
Days to viral suppression median (range)	37.0 (13.0–67.0)	51.5 (26.0–210.0)	176.0 (49.0–1,017.0)	49.0 (13.0–1,017.0)	0.004
Use of supportive services					
Housing	3 (37.5)	7 (41.2)	0 (0.0)	10 (33.3)	0.282
Mental health	3 (37.5)	5 (29.4)	1 (20.0)	9 (30.0)	1.000
Substance use	0 (0.0)	1 (5.9)	0 (0.0)	1 (3.0)	1.000
Insurance	4 (50.0)	3 (17.7)	1 (20.0)	8 (26.7)	0.272
Care coordination	8 (100.0)	14 (56.7)	5 (100)	27 (90.0)	0.732
Visit adherence mean (SD)	0.86 (0.22)	0.91 (0.13)	0.85 (0.16)	0.89 (0.16)	0.715
HIVSS, mean (SD)	96.4 (16.9)	92.1 (15.4)	95.4 (7.7)	93.8 (14.6)	0.771
MMI, mean (SD)	43.4 (1.9)	44.1 (2.8)	44.8 (2.7)	44.0 (2.6)	0.630

^a^Comorbidities included medical diagnoses such as diabetes, hypertension, hyperlipidemia and others with an ICD-10 code, excluding mental health and substance use diagnoses

The median time to linkage to care at our clinic was 9 days, and the median time to viral suppression was 49 days. There was a significant association between time to ART initiation and time to linkage care, and time to ART initiation and time to VLS.

The mean visit adherence over 6 months post-diagnosis was 0.86 among the 0–3 day group, 0.91 among the 4–30 day group, and 0.85 among the > 30 day group, and there was no statistically significant difference between them.

**Fig. 2:**
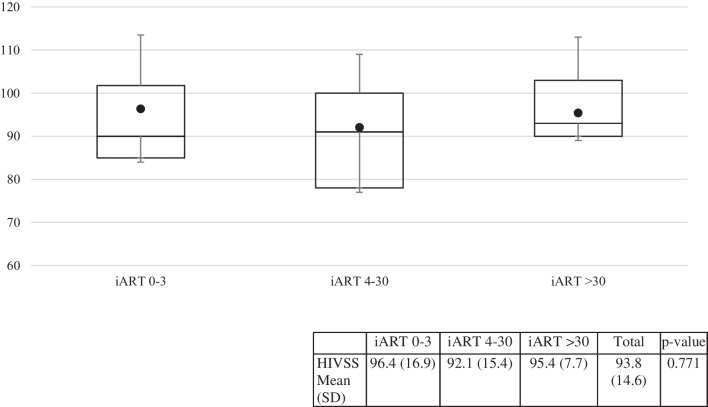
Distribution of HIVSS scores by iART time to initiation

Table 2 shows no statistically significant difference between the mean HIVSS and MMI scores between the groups. Figure 2 shows boxplots of each group’s HIVSS scores at 6 months. The scale items and distribution of each subscale are outlined in Supplement Table S1. The 4–30 day group had the lowest mean HIVSS scores compared to the other groups.

**Fig. 3:**
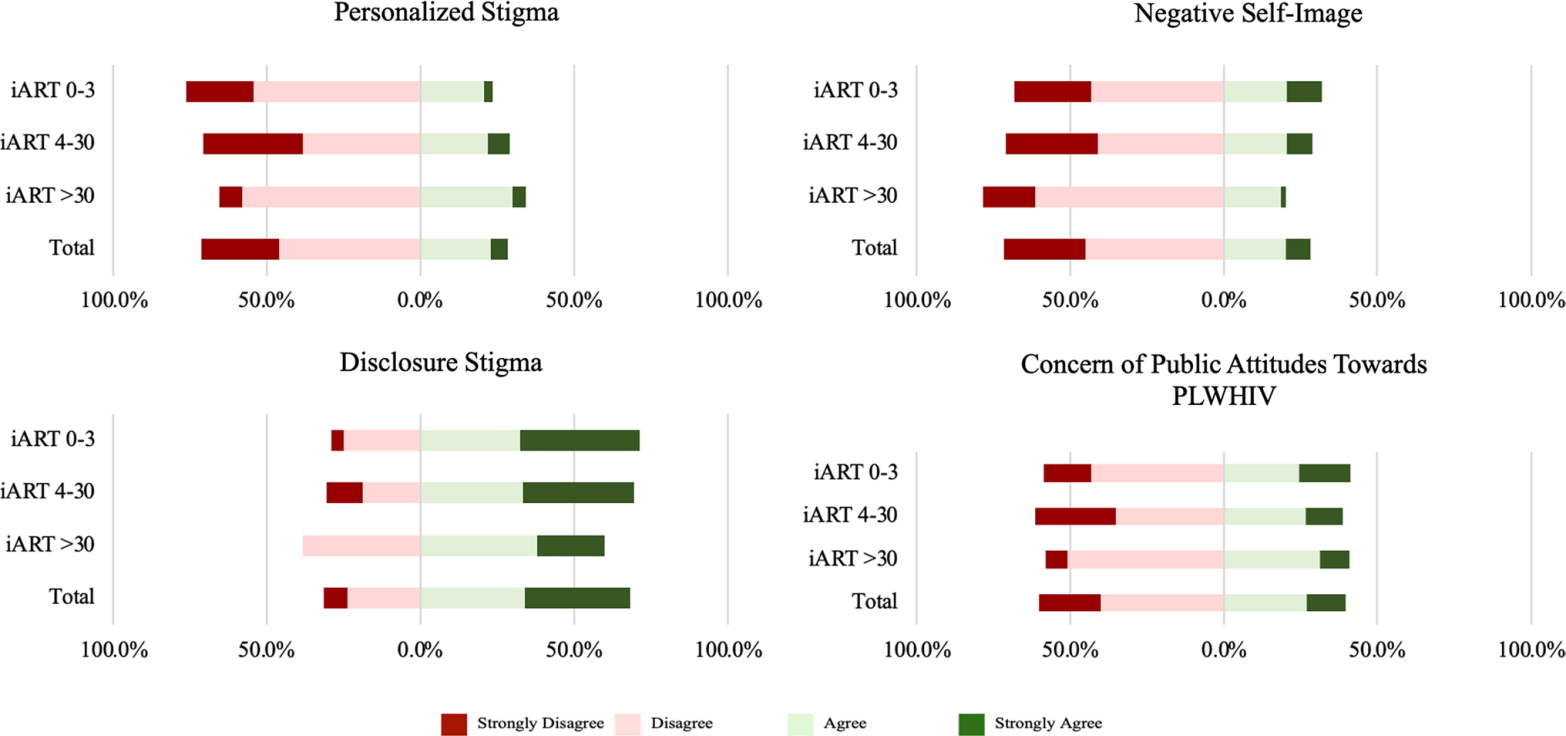
Breakdown of likert results from four sub-scales of the HIVSS by time to ART initiation

Breaking down the HIVSS further into its four subscales corresponding to different types of HIV-related stigma (Fig. 3), the 0–3 day group had the highest negative self-image (e.g., internalized stigma). The > 30-day group had the most personalized stigma (e.g., anticipated, perceived, or enacted stigma) but the least negative self-image or disclosure stigma.

**Fig. 4:**
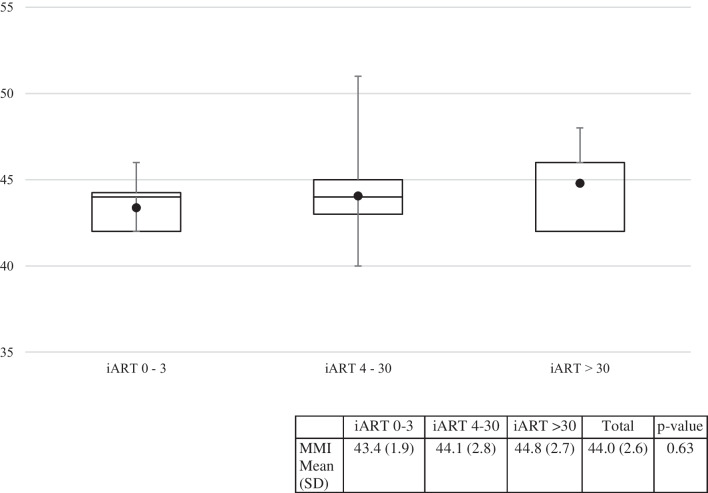
Distribution of MMI scores by iART time to initiation

Figure 4 shows boxplots of the MMI scores for each group at 6 months. The scale items are outlined in Supplement Table S2. The 0–3 day group had the lowest mean MMI score, which increased as time to ART initiation increased.

The results of the sensitivity analysis summarized in Supplement Tables S3 and S4 also showed significant association between time to ART initiation and time to linkage care, and time to ART initiation and time to VLS. The highest mean HIVSS was in the 8–30 day group, the highest mean MMI score was in the > 30 day group, and the highest visit adherence was in the 0–7 day group.

### Qualitative Results

#### “My Beauty wasn’t Going to be Affected”: iART as Stigma Prevention

Participants discussed iART as a means of preventing different stigmatized outcomes they feared after receiving an HIV diagnosis, including *physically appearing sick, having detectable viral load, experiencing discrimination or enacted stigma, and progressing to AIDS and death*. A gay Hispanic male described how his fears after diagnosis resulted from the historical and intersectional stigma of HIV/AIDS with both racism and homophobia at play, and how iART was seen as an opportunity to continue living his life without major change.

“When I was diagnosed, I was just really eager to take medication. The first doctor at CityMD was like ‘It’s not a death sentence.’ When I was diagnosed, the warnings from the ’80s and ‘90s all came back to me. I get they were trying to warn people. Still, it created a huge stigma…during that time when people, even scientists, and doctors, were just bashing the gay community or pinning it on gay people like us, and the minority groups as well…that’s where the stigma comes from, people are scared or nervous about the disease. After I took the medication, I guess I’m somewhat the same person…As long as you take your medication, you’ll be all right.” (Participant 5, 4–30 day ART group, Hispanic Male, Other “Hispanic”)

Immediately following diagnosis, participants voiced their concerns about looking physically ill. Being offered iART provided a way to cope with the possibility of looking sick or physically embodying the disease. A gay Dominican male described how HIV was a threat to his physical appearance and how iART helped combat that process.

“I feel like [iART] played a huge role. I felt like it was gonna keep me healthy. You weren’t gonna see it on me. My beauty wasn’t going to be affected which was very important to me. I had a very positive experience with starting the antiretrovirals.” (Participant 22, 0–3 day ART group, Hispanic Male, Other “Dominican”)

Many participants described not remembering what was said about the medication after being diagnosed. A white gay Hispanic male discussed not knowing what was in ART when it was offered due to the shock of just receiving an HIV diagnosis but believing it would help him avoid deteriorating health and give him an opportunity at a productive life.

“People with HIV have a really difficult life, not because of the sickness itself, but how their lives change-their career, their future, their relationships, their families, they become isolated. They don’t have a chance to succeed… When they told me that I was HIV positive, your mind goes to all those memories, and, you say, I’m gonna take the pill. I’m gonna do whatever it takes to be as normal as possible. In order not to get sick, not look too skinny, you have all those fears, people are gonna find out that I’m HIV positive. You don’t really know what it’s doing to your body.” (Participant 4, 4–30 day ART group, Hispanic, Male, Other “Hispanic/Latino”)

Participants also discussed concerns about having an elevated viral load and passing HIV to a future partner. iART allayed those fears, applying the well-known treatment as prevention (TasP) concept to stigma prevention. A gay white male discussed how iART decreased his worry.

“There is such a stigma around people that have HIV that I wanted to make sure that I was undetectable. I wanted to make sure that I wasn’t going to pass on this virus and that I could live a normal life. I think that the stigma propelled me to want to take the medication so I didn’t worry about what others thought about me in their sexual life.” (Participant 9, 4–30 day ART group, Non-Hispanic, Male, White)

One participant, a recent immigrant, described the difference with iART in the U.S. versus what they believed it would be in their home country.

“I come from the third-world and coming to a first-world level where HIV is considered a chronic illness and not a deadly one, is a tremendous change. Where you have a doctor who explains to you that your illness doesn’t have a stigma or isn’t a death sentence versus having a doctor who quite possibly wouldn’t even discuss treatment.” (Participant 7, 4–30 day ART group, Hispanic Male, Other “Latino”)

While iART felt empowering in preventing future stigma, some participants described the emotional challenge of choosing between life and death. Many were disengaged from the details of their treatment as a result. One participant lamented the feeling of having no choice but to start immediately because the alternative meant the end of their life.

“I didn’t have a choice…It was, all right, this is what I have to do in order for me to live. Like I wanna live, so I gotta take the pill. Simple as that. I don’t want it to turn into AIDS. I’d rather be undetectable than almost die from it.” (Participant 29, 4–30 day ART group, Non-Hispanic Male, Black/African American)

#### “The Stigma Tells you one Thing, but Living with it Means Something Different”: iART Alleviating Internalized Stigma

The stigma at the time of diagnosis was often internalized and intersectional for participants of varied identities, which added to levels of anxiety about what a new HIV diagnosis would mean for their lives. One participant described how being gay complicated his coping with an HIV diagnosis.

“I mean, if I were straight, it’d be a different story, but because I’m gay, it’s like, ‘Oh, he’s gay. He got HIV. Surprise, surprise.’ I hate that feeling. What we’re taught by nature through schooling, through society is that it’s a gay curse. It’s a gay virus. That’s not okay. It kind of sucks that I am gay and that I got it because it’s almost affirming that.“ (Participant 9, 4–30 day ART group, Non-Hispanic Male, White)

iART provided a mechanism for coping with this kind of internalized, intersectional stigma defined by homophobia in the community and the sexual stigma of HIV. Another participant recalled:

“I almost feel like my race and my sexual identification added to the stigma instead of decreased the stigma…The stigma around HIV has always had a heavy weight for people of color and gay people. [When I was diagnosed], it was presented as take a pill a day and your life just blossoms. So it removed a lot of stigma…because [iART] was presented as ‘This is going to help you keep HIV down so it doesn’t get in the way of living. The stigma tells you one thing, but living with it means something completely different…so getting the medication quickly helped lessen a lot of my own personal feelings of stigma.”(Participant 10, 0–3 day ART group, Hispanic Male, Other “Latin American”)

Many participants described the internal turmoil after HIV diagnosis, often reporting feeling disappointed in themselves. The immediate offer of ART served to re-focus these negative feelings into action. Participant 28 discussed how the process of psychological self-destruction was disrupted by iART.

“I’ve never really faced any kind of discrimination. So if there’s any discrimination, it’s on my part. I personally trash talked myself, was real harsh with myself for where I ended up. I think that’s what motivated me to start [iART]. It was a good feeling in a disappointing moment. Getting diagnosed was not something I wanted to ever fall into. But I was really happy there was something I could use to keep me going, that immediately they gave me something.” (Participant 28, 0–3 day ART group, Non-Hispanic Male, Black/African American)

Many participants described the internalized stigma resulting from the transition from being a “healthy” person to being a “sick” person. However, iART reframed this dichotomous paradigm for some participants. Participant 25 described how iART helped them realize that because they now were living with HIV didn’t mean they were necessarily “sick”.

“It helps you live beyond the stigma because you know that when you start taking it, you are taking something to better your situation and correct whatever is going on so that you are not going to get sick. Having HIV doesn’t mean you’re sick.” (Participant 25, 0–3 day ART group, Non-Hispanic Male, Black/African American)

#### “I was Looked Upon Differently”: iART Exacerbating Anticipated or Perceived Stigma

Many participants stressed that while iART was helpful with the internalized stigma, the offer of medication was different from actually picking up and initiating the medications, which brought new stresses and feelings of stigma. The pharmacy was a source of anticipated or perceived stigma among participants and many recalled their struggles with their first visit.

“[Going to the pharmacy] took me a little bit. I used to have my partner go and get my medication because I felt a little embarrassed, but I got over it and I was able to start picking up my own medications sometimes.” (Participant 22, 0–3 day ART group, Hispanic Male, Other “Dominican”)

“I didn’t get them the first day because I was afraid. I went in the second day and said no I’m not gonna risk my health…[My initial fear] was how people were gonna react at the pharmacy.” (Participant 16, > 30 day ART group, Non-Hispanic Male, Black/African American)

“I would go when nobody was in the pharmacy. Or I would tell my dad to pick up my medicine.” (Participant 8, 0–3 day ART group, Hispanic Woman, Black/African American)

While these participants discussed anticipated stigma, other participants who did go to pick up iART reported the perception of being looked down upon by pharmacy staff. One participant from abroad was surprised to find this dynamic in the U.S.

“At the pharmacy, at first, I was ashamed when I had to go to get the medicines. Everybody looks at you when you go to the pharmacy... I thought that in this country it was different because it is such a developed country, but even here they still look at you.” (Participant 24, > 30 day ART group, Hispanic Male, Other “Latino”)

A Black woman with other co-morbidities compared the experience of picking up her ART to picking up other medications for her other chronic diseases.

“Oh it made me feel like I was looked upon differently. Just for that moment, it made me feel a little ashamed. Because everything else I would come in there for was basically for the liver cirrhosis, and the stroke medication. So, he saw the [ART], I just felt like I was being judged. Emptiness, a little loneliness. Just felt sad for a moment... And I noticed that one person, I guess the pharmaceutical tech noticed that I was taking the antiviral medicine and just the look when he saw it. It was just that non-verbal communication. He looked at me differently.” (Participant 2, 4–30 day ART group, Non-Hispanic Woman, Black/African American)

While discussing how starting immediately may have brought up anticipated or perceived stigma, participants disaggregated the immediate offer (the ‘i’) from the medication itself (the ‘ART’). While many had a positive perception of iART being offered right away, they had a negative perception of the physical ART pill, which was a source of stigma. Participant 7 described how the medications were a constant reminder of his HIV:

“At first when I would take the medication I would feel dirty. I would tell myself “You’re contaminated.” In the beginning taking the medication every morning was the most difficult thing, knowing I had the virus, it was harder to have the medication in hand” (Participant 7, 4–30 day ART group, Hispanic Male, Other “Latino”)

Another described feeling that their entire identity was reduced to having HIV due to having to take medication every day. For participant 9, this initial stigma abated over time.

“I felt like I was the disease in a sense because I was having to take this obligatory one pill a day. I thought, ‘Oh my God, I am the disease.’ I take this pill everyday. It’s such a burden. But now it’s like anyone else who has to take medications.” (Participant 9, 4–30 day ART group, Non-Hispanic Male, White)

While iART alleviated internalized feelings of stigma, it did not remove the stigma associated with taking the medication and the anticipation of a negative reaction from anyone who would see them. Participant 4 discussed the various places where he is preoccupied with hiding his medication, so it is not incidentally found.

I have to make sure that no one is gonna see the pills, that no one is gonna check my backpack…You become paranoid when you have to leave your house, or if you have visitors. If you have the pills in your bathroom, you need to make sure to hide those pills so no one sees the label.” (Participant 4, 4–30 day ART group, Hispanic Male, Other “Hispanic/ Latino”)

#### “It’s the Foundation of the Relationship”: iART Establishing Trust in Provider

Participants described iART as the basis of trust with their HIV care provider. They believed having a shared focus on the decision to start medication and future goals brought them closer to understanding and having confidence in their provider’s motivations. Participant 2 described the impact of iART on the relationship.

“Taking that medication so fast, it allowed my relationship with my providers there to be built from the ground up. It’s the foundation of the relationship. It’s about the medication. We hit the ground rolling with it so it was just like any other medication…And that allowed me to open up more too. I just felt safe.” (Participant 2, 4–30 day ART group, Non-Hispanic Woman, Black/African American)

However, iART often reminded participants of nefarious profit motives of the healthcare system, which included drug stores, pharmaceutical companies, large hospitals, and other entities they saw as profiting. In this context, the iART emphasis on immediate medication created concern that treatment recommendations were driven by motivations other than patient health. Participant 4 described their experience choosing to start medication while not trusting the health system.

“I was reading about these companies that make a lot of money from making HIV treatment for people like me, and some [patients] who cannot really afford it, or don’t have health insurance. These companies, they don’t care…You see a lot of news, and from friends and relatives experiences where the health system just failed so badly. For my specific case, I’m just doing what I’ve been told. And, it’s awful when you think about it, you don’t even know what you’re putting inside your body with those pills. But, it’s [start] taking the medication, or face even worse consequences.” (Participant 4, 4–30 day ART group, Hispanic Male, Other “Hispanic/Latino”)

Participants also identified a shared-decision making approach as an essential component of building trust through iART. Participant 12 identified shared decision-making as key to building mutual respect through the first major decision for provider and patient.

“I would recommend that when seeing patients like myself, especially in a clinic where a lot of the doctors are white males and treating a lot of patients that look like myself in the hospital. I would say what my doctor did, when he laid out the different options in front of me, that was very comforting, I liked that a lot…Even though he’s the one with the power, it feels more so like a dialogue and how we can both move forward.” (Participant 12, > 30 day ART group, Hispanic Male, Other “Mixed”)

Participants emphasized that trust is a process rather than an immediate feeling. Some discussed coming in actively distrusting the healthcare system but then seeing how interpersonal relationships made an impact longitudinally. Participant 7 discussed how this process evolved through iART.

[iART] is very very effective. I didn’t think the group of doctors would be this prepared, it was practically immediate. I was diagnosed between 10 and 11pm on Thursday or Friday, and started treatment on Saturday… In the beginning, [my trust] was very low. You go in there thinking they don’t care what I am going through. As you come back every month, every three months, that changes. They let you know that they are here for you.” (Participant 7, 4–30 day ART group, Hispanic Male, Other “Latino”)

#### “I Wanted to see my Progress”: iART as Motivation for Care Engagement

For most participants, starting ART immediately served as motivation to stay engaged in HIV care. Participants defined their engagement in different ways, some said it was about physically coming to clinic and taking their daily medication. Participant 7 described iART being the first step in a process in which they begin to learn how to live with HIV.

“It’s a process and it’s a bodily change, both physical and psychological. Thank God, every month I had to be at the clinic. It was a great source of support because you go through many changes, hormonal and physical. And having someone who constantly explains what you’re going through is surreal. *Because you don’t feel alone.* So you start your medication and you come every month, every three months to an appointment with your doctor. To me [being engaged] is visiting the clinic and taking your medication.” (Participant 7, 4–30 day ART group, Hispanic Male, Other “Latino”)

iART also served as the beginning of seeing progress in viral load suppression and CD4 count recovery, as recounted by Participant 12.

“But taking the medications made me actually more eager to go to the doctors because I wanted to see my progress. I wanted to reach the milestone of being undetectable. And after I reached that milestone, I look forward to going to the doctor every time to make sure that I am still undetectable (Participant 12, > 30 day ART group, Hispanic Male, Other “Mixed”)

However, physically coming to appointments exposed many participants to stigma, particularly in the waiting room. Participant 2 discussed struggling with the lack of privacy involved in coming to the clinic.

“I just don’t want people to know me or hear my name, see me inside of [the clinic]. I want to keep the anonymity there. I would like to be more private. That’s all.” (Participant 2, 4–30 day ART group, Non-Hispanic Woman, Black/African American”)

Participant 20 recalled the mental anguish of seeing PWH struggling in the waiting room and that representing a potential fate they would meet.

“[Seeing] the people that are having a harder experience than me makes it hard. I can’t really be in the waiting room that long. The waiting room is not a good experience for me, mentally…I just get a little scared to see very sick people in the same clinic as I am.” (Participant 20, 0–3 day ART group, Hispanic Male, Other “Dominican”)

## Integrated Quantitative and Qualitative Results

Table 3 shows integrated findings from quantitative and qualitative results. Identified themes that were present in the majority of cases in each group were included.

**Table 3:** Integrating iART, themes, representative quotes, HIVSS, MMI, and visit adherence at 6 months

	Themes	Representative quotes	Mean HIVSS score	Mean MMI score	Mean visit adherence
0–3 days			96.4 (16.9)	43.8 (1.9)	0.86 (0.22)
	iART as stigma prevention^a^	My cousin and his wife were heroin addicts. They were a beautiful couple…They found out they had HIV, it was the early 80s. Everybody had that stigma. And they didn’t live long…I was offered [ART] when I went to speak to my doctor, she sent me straight to the ER. And an HIV specialist told me what was available, I think I took two pills. I felt hopeful. It was promising, to know that I was able to get my viral load under control – Participant 3			
4–30 days			92.1 (15.4)	44.1 (2.8)	0.91 (0.13)
	Alleviation of Internalized stigma^b^	You know people who got HIV have a really difficult life. Not because of the sickness itself, but around their lives, everything changes. Their career, their future, their relationship with their families. They become, isolated. They don’t have a chance to succeed. Maybe that’s the image I got when I was young. And, then, when they told me that I was HIV positive, of course, your mind goes to all those memories. I said, okay, I’m gonna take the pill. I’m gonna do whatever it takes in order to be as normal as possible. -Participant 4			
30 + days			95.4 (7.7)	44.8 (2.7)	0.85 (0.16)
	Exacerbation of perceived or anticipated stigma^c^	At the pharmacy, at first, I was ashamed when I had to go to get the medicines. Everybody looks at you when you go to the pharmacy if there are new employees… I thought that in this country it was different because it is such a developed country, but even here they still look at that. Even at my job, that hasn’t happened to me. -Participant 24			
Total			93.8 (14.6)	44.0 (2.6)	0.89 (0.16)

^a^This theme was found in 5 out of the 8 participants

^b^This theme was found in 10 out of the 16 participants

^c^This theme was found in 3 out of the 5 participants

The 0–3 day group had the highest mean HIVSS score, the lowest MMI score, and a lower visit adherence than the overall mean. iART as stigma prevention was the theme unique to this group. This group emphasized that iART mitigated community and anticipated forms of stigma, and had the lowest personalized stigma subscale score, yet this group still had the highest negative self-image subscale score.

The 4–30 day group had the lowest mean HIVSS score and the highest visit adherence. Alleviation of internalized stigma was the theme unique to this group. This group emphasized that iART actively reduced internal feelings of shame, and this group had a lower negative self-image subscale score than the 0–3 day group, meaning iART potentially reduced their stigma scores 6 months post-diagnosis.

The > 30-day group had a higher mean HIVSS score, the highest MMI score, and a lower visit adherence than the overall mean. Exacerbation of perceived or anticipated was the theme unique to this group. This group emphasized that iART perhaps worsened anticipation or perception of stigmatizing experiences, meaning that iART potentially increased their stigma scores 6 months post-diagnosis. The > 30 day group had the highest personalized stigma subscale score (e.g., anticipated, perceived, or enacted stigma), which is consistent with the qualitative data.

The themes of Foundation of Provider Trust and iART as care motivation were equally present in all three groups.

## Discussion

This is the first mixed-methods study to explore the impact of iART on the important psychosocial constructs of stigma and medical trust/mistrust, as well as longitudinal HIV care engagement. This builds on recent studies examining barriers to and facilitators of rapid ART to further explore the impact of iART on newly diagnosed PWH.

This study demonstrates the importance of a mixed-methods approach. Electronic medical chart review is often inadequate in capturing important demographic variables when considering HIV care inequities and capturing the complex process from diagnosis to linkage to ongoing care. Our approach allowed us to triangulate our data to have the most accurate understanding of each participant’s case. Furthermore, we contextualized our quantitative measures of levels of stigma and mistrust with qualitative data that illuminated the longitudinal psychosocial processes that occurred after ART initiation.

This study also begins to characterize HIV care trajectories based on timing of ART initiation. It illuminates the consequential role of HIV-related stigma for newly diagnosed PWH. For the group that started ART same-day or within 72 h, iART appeared to mitigate the development of anticipated or enacted stigma, and community stigma. Significant advancements in the effectiveness of ART empowered participants to feel they could avoid future stigmatizing experiences if they started ART immediately. Because this did not necessarily lead to greater mean visit adherence than other groups, other mediators or systemic factors were likely at play when it came to long-term HIV care engagement for this group.

For the 4–30 day group, iART helped alleviate internalized HIV stigma. Participants coming in feeling shame about their diagnosis were able to process their emotions through starting iART, providing an opportunity for coping with and reducing the internalized HIV stigma built up over time. This group also had the highest visit adherence, possibly suggesting that the active alleviation of these feelings made this group more comfortable keeping their appointments. Future iART implementation may include a stigma intervention along with other social determinants of health that are part of the iART package (e.g. housing, insurance).

Participants who took the longest to initiate ART (> 30 days) specifically highlighted the role of anticipated, perceived, or enacted stigma. The ART in iART was the first manifestation or reminder of one’s HIV status in a vulnerable time after diagnosis, which led participants to hide medications or highlight difficult experiences picking up ART at the pharmacy. This group also had the highest level of mistrust and a lower visit adherence. This provides a ripe area for future study and interventions, such as enhanced peer support with the pharmacy or more pharmacist education regarding iART and stigma reduction.

Within every group, participants emphasized that iART was an opportunity to build trust and engage with their provider. The concept of shared decision-making, mainly when participants felt they did not choose whether or not to initiate ART, was particularly stressed. This could be a critical concept in educating providers and clinics on how to implement iART successfully. Furthermore, scores from the MMI, which increased with time to ART initiation, may point to an opportunity to engage with feelings of mistrust with the health system and medical establishment at diagnosis.

## Limitations

There were a few limitations to this study. First, all participants were retained in care. Given that we recruited in the clinical setting, it was challenging to recruit patients who had fallen out of care. This likely introduced selection bias to our results, missing the crucial perspectives of those lost to follow-up or not consistently in care. In addition, our study was retrospective rather than prospective. Without a pre-iART baseline, we cannot make any definitive conclusions about the effect of iART on HIVSS and MMI scores, and vice versa. Ideally, we would also want to know how scores from the MMI and HIVSS surveys at the time of diagnosis/iART change over time. We utilized the qualitative data, in which participants describe the process from pre-ART initiation to 6 months post-ART initiation, to infer how these scores may have changed, however this is a pilot using cross-sectional data to generate hypotheses and establish areas of future inquiry. Lastly, this study only covered 6 months visit adherence, and likely a longer period of observation would yield more pertinent data on HIV care engagement over time.

## Future Directions

Future directions include a focus on PWH who are out of care, as they are expected to receive the same iART intervention around the country. Participants who initiated iART > 30 days may share features with those out of care and merit further exploration. Implementation science will be critical to identify other important barriers or facilitators to iART given that iART will be carried out in emergency rooms, sexual health clinics, community organizations, etc. Successful iART implementation will encompass entire hospital systems and community networks. As mentioned above, future studies may use a behavioral science approach to measure longitudinal changes in HIVSS, MMI and other psychometric surveys before HIV diagnosis, at diagnosis, and after diagnosis to better understand associations with iART and HIV care engagement. Lastly, it will be essential to leverage technology and virtual care to optimize iART implementation with the repeated disruptions during the COVID-19 pandemic.

## Conclusion

This mixed-methods pilot study is the first to provide crucial context to the psychosocial costs and benefits of iART. In terms of care trajectories, iART likely does not overcome established systemic forces that lead to patient disengagement in care. Still, in our study, this population laid out the benefits of iART when done well. Universal approaches to iART may be coupled with an equitable implementation strategy that addresses the deleterious impact of stigma, incorporates concerns around mistrust, and other psychosocial factors that resonate with the communities most impacted by HIV. Identifying patients who need focused support (e.g., support at the pharmacy picking up medication for the first time) and training providers to practice iART successfully (e.g., highlighting shared decision making) will be paramount to optimal iART implementation.

### Supplementary Information

Below is the link to the electronic supplementary material.

Supplementary file1 (DOCX 27 KB)

## Data Availability

Not applicable.

## Appendix

See Tables 4 and 5.

**Table 4:** Designation of HIVSS items to four sub-scales: personal stigma, disclosure, negative self-image, public attitudes

#	Item	Subscale
1	In many areas of my life, no one knows I have HIV	2
2	I feel guilty because I have HIV	3
3	People’s attitudes make me feel worse about myself	3
4	Telling someone I have HIV is risky	2, 4
5	People with HIV lose jobs when employers learn about their HIV status	4
6	I work hard to keep my HIV status a secret	2, 3
7	I feel I’m not as good as others because I have HIV	3
8	I never feel ashamed of having HIV (R)	3
9	People with HIV are treated like outcasts	4
10	Most people believe a person who has HIV is dirty	4
11	It is easier to avoid friendships than to worry about telling people about my HIV status	2, 3, 4
12	Having HIV makes me feel unclean	3
13	I feel set apart, isolated from the rest of the world	1, 3, 4
14	Most people think a person with HIV is disgusting	4
15	Having HIV makes me feel I’m a bad person	3
16	Most people with HIV are rejected when others learn about their status	1, 4
17	I am very careful with whom I tell that I have HIV	2
18	Some people who know about my HIV status have grown more distant	1
19	I worry about people discriminating against me	2, 4
20	Most people are uncomfortable around someone with HIV	4
21	I never feel I have to hide the fact that I have HIV (R)	2
22	I worry that people may judge me when they learn about my HIV status	2, 4
23	Having HIV in my body is disgusting to me	3
24	I am hurt by how people react when they learn I have HIV	1
25	I worry people who know I have HIV will tell others	2
26	I regret having told some people that I have HIV	1
27	As a rule, telling other has been a mistake	1, 3, 4
28	People avoid touching me if they know I have HIV	1, 4
29	People I care about stopped calling me after learning that I have HIV	1
30	Some people told me that HIV is what I deserved for how I’ve lived	1, 4
31	Some people close to me are afraid others will reject them if it becomes known I have HIV	1
32	People don’t want me around their children once they know I have HIV	1, 4
33	People have physically backed away from me when they know I have HIV	1, 4
34	Some people act as though it’s my fault I have HIV	1, 4
35	I have stopped socializing with some people because of their reactions to my having HIV	1
36	I have lost friends by telling them I have HIV	1
37	I have told people close to me to keep my HIV secret	2
38	People who know tend to ignore my good points	1, 3, 4
39	People seem afraid of me because I have HIV	1, 3, 4
40	Knowing you have HIV; they look for flaws in your character	1, 4

1—Personalized stigma 2—Disclosure 3—Negative self-image 4—Public attitudes

(R) Indicates an item is reverse scored

**Table 5:** MMI items

#	Item
1	You’d better be cautious when dealing with healthcare organizations
2	Patients have sometimes been deceived or mislead by healthcare organizations
3	When healthcare organizations make mistakes they usually cover it up
4	Healthcare organizations have sometimes done harmful experiments on patients without their knowledge
5	Healthcare organizations don’t always keep your information totally private (R)
6	Sometimes I wonder if healthcare organizations really know what they are doing
7	Mistakes are common in healthcare organizations (R)
8	I trust that health care organizations will tell me if a mistake is made about my treatment (R)
9	Health care organizations often want to know more about your business than they need to know (R)
10	The patient’s medical needs come before other considerations at health care organizations (R)
11	Health care organizations are more concerned about making money than taking care of people (R)
12	Health care organizations put the patient’s health first
13	Patients should always follow the advice given to them at health care organizations
14	I typically get a second opinion when I am told something about my health
15	I trust that health care organizations check their staff’s credentials to make sure they are hiring the best people
16	They know what they are doing at health care organizations
17	I trust that health care organizations keep up with the latest medical information

(R) Indicates an item is reverse scored
