# Supplementary material for: Rapid or Immediate ART, HIV Stigma, Medical Mistrust, and Retention in Care: An Exploratory Mixed Methods Pilot Study
Source: AIDS Behav. 2023 Apr 18;27(10):3430–46. doi: 10.1007/s10461-023-04058-4 (PMC10111080; doi:10.1007/s10461-023-04058-4)
Supplement: Supplementary file 1 — Supplementary file1 (DOCX 27 KB) [file 10461_2023_4058_MOESM1_ESM.docx]

**Table S1. Demographic Characteristics of Participants – iART defined as within 7 days (N=30)**

|  | Time to ART initiation (N=30) | | |  | |
| --- | --- | --- | --- | --- | --- |
|  | 0-7  (N=19) | 8-30  (N=6) | >30  (N=5) | Total (N=30) | p-value |
| Characteristic | N (%) | N (%) | N (%) | N (%) |  |
| Age |  |  |  |  | 0.432 |
| Mean (SD) | 35.9  (9.5) | 30.3  (5.2) | 39.4  (22.1) | 35.4  (11.7) |  |
| Country of Birth |  |  |  |  | 1.000 |
| United States/territory | 14 (73.7) | 5 (83.3) | 4 (80.0) | 23 (76.7) |  |
| Other | 5 (25.3) | 1 (16.7) | 1 (20.0) | 7 (23.3) |  |
| Primary Language Spoken |  |  |  |  | 0.803 |
| English | 14 (73.7) | 5 (83.3) | 3 (60.0) | 22 (73.3) |  |
| Spanish | 4 (21.1) | 1 (16.7) | 2 (40.0) | 7 (23.3) |  |
| Other | 1 (5.3) | 0 (0.0) | 0 (0.0) | 1 (3.3) |  |
| Race* |  |  |  |  | **0.010** |
| Black/African American | 7 (36.8) | 1 (16.7) | 2 (40.0) | 10 (33.3) |  |
| Native American/ Alaskan Native | 0 (0.0) | 1 (16.7) | 0 (0.0) | 1 (3.3) |  |
| White | 0 (0.0) | 3 (50.0) | 0 (0.0) | 3 (10.3) |  |
| Other | 12 (63.2) | 1 (16.7) | 3 (60.0) | 16 (53.3) |  |
| Ethnicity |  |  |  |  | 0.318 |
| Hispanic | 13 (68.4) | 2 (33.3) | 3 (60.0) | 18 (60.0) |  |
| Non-Hispanic | 6 (31.6) | 4 (66.7) | 2 (40.0) | 12 (40.0) |  |
| Gender Identity |  |  |  |  | 0.869 |
| Man | 14 (73.6) | 5 (83.3) | 5 (100) | 24 (80.0) |  |
| Woman | 4 (21.1) | 1 (16.7) | 0 (0.0) | 5 (16.7) |  |
| Transgender Woman | 1 (5.3) | 0 (0.0) | 0 (0.0) | 1 (3.3) |  |
| Sexual Orientation |  |  |  |  | 0.817 |
| Heterosexual/ Straight | 6 (31.6) | 1 (16.7) | 3 (60.0) | 10 (33.3) |  |
| Bisexual | 1 (5.3) | 1 (16.7) | 0 (0.0) | 2 (6.7) |  |
| Gay | 10 (52.6) | 4 (66.7) | 2 (40.0) | 16 (53.3) |  |
| Queer | 1 (5.3) | 0 (0.0) | 0 (0.0) | 1 (3.3) |  |
| Unsure/Questioning | 1 (5.3) | 0 (0.0) | 0 (0.0) | 1 (3.3) |  |
| Highest level of Education |  |  |  |  | 0.521 |
| Some High School | 2 (10.5) | 0 (0.0) | 0 (0.0) | 2 (6.7) |  |
| High School Diploma/GED | 2 (10.5) | 2 (33.3) | 2 (40.0) | 6 (20.0) |  |
| Associate’s Degree/Some College | 9 (47.4) | 1 (16.7) | 2 (40.0) | 12 (40.0) |  |
| Bachelor’s Degree or Higher | 6 (31.6) | 3 (50.0) | 1 (20.0) | 10 (33.3) |  |
| Employment Status |  |  |  |  | 0.227 |
| Employed/Self-employed | 9 (47.4) | 4 (66.7) | 1 (20.0) | 14 (46.7) |  |
| Unemployed/Unable to work | 9 (47.4) | 2 (33.3) | 2 (40.0) | 13 (43.3) |  |
| Student/Retired | 1 (5.3) | 0 (0) | 2 (40.0) | 3 (10.0) |  |
| Housing Situation |  |  |  |  | 0.607 |
| Stably Housed | 18 (94.7) | 5 (83.3) | 5 (100) | 28 (93.3) |  |
| Unstably Housed | 1 (5.3) | 1 (16.7) | 0 (0) | 2 (6.7) |  |
| Current Annual Income |  |  |  |  | 0.214 |
| <$20,000 | 8 (42.1) | 2 (33.3) | 3 (60.0) | 13 (43.3) |  |
| $20,000-39,999 | 4 (21.1) | 0 (0.0) | 2 (40.0) | 6 (20.0) |  |
| $40,000-59,999 | 4 (21.1) | 4 (66.7) | 0 (0.0) | 8 (26.7) |  |
| >$60,000 | 3 (15.8) | 0 (0.0) | 0 (0.0) | 3 (10.0) |  |
| Health Insurance** |  |  |  |  | 0.803 |
| ADAP | 2 (10.5) | 1 (16.7) | 0 (0.0) | 3 (10.0) |  |
| Medicaid/Medicare | 10 (52.6) | 4 (66.7) | 4 (80.0) | 18 (60.0) |  |
| Private | 7 (36.8) | 1 (16.7) | 1 (20.0) | 9 (30.0) |  |
| Marital Status |  |  |  |  | 0.483 |
| Never Married | 13 (68.4) | 4 (66.7) | 2 (40) | 19 (63.3) |  |
| Divorced/Separated/ Other | 6 (31.6) | 2 (33.3) | 3 (60) | 11 (36.7) |  |

*Preference was given to patient survey responses over chart review when determining race and ethnicity. All responses of “Other” in which “Latino,” Hispanic,” or some variant were entered were maintained as “Other” as per NIH guidelines (patients also responded “Puerto Rican” “Spanish” and “Dominican”; one patient said “mixed” and one said “biracial”)

**PLWH who are uninsured or underinsured in New York state are eligible for immediate coverage via ADAP (AIDS Drug Assistance Program). Patients who present to our site without insurance are enrolled immediately, so no patient in this cohort is listed as uninsured.

**Table S2. Clinical Characteristics of Participants – iART defined as within 7 days (N=30)**

|  | Time to ART Initiation (Days) | | |  | |
| --- | --- | --- | --- | --- | --- |
|  | 0-7  (N=19) | 8-30  (N=6) | >30  (N=5) | Total  (N=30) | p-value |
| Characteristic | N (%) | N (%) | N (%) | N (%) |  |
| HIV Risk Factor |  |  |  |  | 0.435 |
| Heterosexual sex | 6 (31.6) | 1 (16.7) | 2 (40.0) | 9 (30.0) |  |
| MSM | 12 (63.2) | 5 (83.3) | 2 (40.0) | 19 (63.3) |  |
| MSM+IDU | 1 (5.3) | 0 (0.0) | 0 (0.0) | 1 (3.3) |  |
| IDU | 0 (0.0) | 0 (0.0) | 1 (20.0) | 1 (3.3) |  |
| Number of Comorbidities* |  |  |  |  | 0.051 |
| 0-2 | 16 (84.2) | 6 (100) | 2 (40.0) | 24 (80.0) |  |
| 3-5 | 2 (10.5) | 0 (0.0) | 3 (60.0) | 5 (16.7) |  |
| 6+ | 1 (5.3) | 0 (0.0) | 0 (0.0) | 1 (3.3) |  |
| Mental Health Diagnosis |  |  |  |  | 0.493 |
| Yes | 6 (31.6) | 2 (33.3) | 0 (0.0) | 8 (26.7) |  |
| No | 13 (68.4) | 4 (66.7) | 5 (100) | 22 (73.3) |  |
| Substance Use Diagnosis |  |  |  |  | 0.486 |
| Yes | 6 (31.6) | 1 (16.7) | 0 (0.0) | 7 (23.3) |  |
| No | 13 (68.4) | 5 (83.3) | 5 (100) | 23 (76.7) |  |
| Initial CD4 |  |  |  |  | 1.000 |
| >200 | 15 (78.9) | 5 (83.3) | 4 (80.0) | 24 (80.0) |  |
| <200 | 4 (21.1) | 1 (16.7) | 1 (20.0) | 6 (20.0) |  |
| Initial Viral Load |  |  |  |  | 0.448 |
| < 100,000 | 9 (47.4) | 2 (33.3) | 3 (60.0) | 14 (46.7) |  |
| 100-500,000 | 4 (21.1) | 3 (50.0) | 2 (40.0) | 9 (30.0) |  |
| >500,000 | 6 (31.6) | 1 (16.7) | 0 (0.0) | 7 (23.3) |  |
| Days to Linkage to Care  Median (range) | 5.0  (0.0-210.0) | 12.0  (4.0-131.0) | 146.0  (35.0-163.0) | 9.0  (0.0-210.0) | **0.007** |
| Days to Viral Suppression  Median (range) | 44.0  (13.0-210.0) | 70.0  (31.0-131.0) | 176.0  (49.0-1,017.0) | 49.0  (13.0-1,017.0) | **0.022** |
| Use of Supportive Services |  |  |  |  |  |
| Housing | 8 (42.1) | 2 (33.3) | 0 (0) | 10 (33.3) | 0.283 |
| Mental health | 7 (36.8) | 1 (16.7) | 1 (20.0) | 9 (30.0) | 0.622 |
| Substance use | 1 (5.3) | 0 (0.0) | 0 (0.0) | 1 (3.3) | 1.000 |
| Insurance | 6 (31.6) | 1 (16.7) | 1 (20) | 8 (26.7) | 0.851 |
| Care coordination | 17 (89.5) | 5 (83.3) | 5 (100) | 27 (90) | 1.000 |
| Visit Adherence  Mean (SD) | 0.91  (0.16) | 0.85  (0.16) | 0.85  (0.15) | 0.89  (0.16) | 0.642 |
| HIVSS  Mean (SD) | 91.4  (15.2) | 99.8  (16.7) | 95.4  (7.7) | 93.8  (14.6) | 0.466 |
| MMI  Mean (SD) | 43.8  (2.0) | 43.8  (4.1) | 44.8  (2.7) | 44.0  (2.6) | 0.759 |
